# Caste, local governance effectiveness, and multidimensional poverty in rural India: some evidences

**DOI:** 10.3389/fsoc.2025.1482825

**Published:** 2025-02-11

**Authors:** Amarachi Onyeyirichi Ogbonna, Charles Aaron Adams Ekuban, C. Muralee Krishnan, P. K. Viswanathan

**Affiliations:** ^1^Amrita School for Sustainable Futures, Amrita Vishwa Vidyapeetham, Amritapuri, India; ^2^Amrita School of Business, Amrita Vishwa Vidyapeetham, Coimbatore, India; ^3^Amrita School of Business, Amrita Vishwa Vidyapeetham, Amritapuri, India

**Keywords:** multidimensional poverty, local governance effectiveness, SC/ST population density, poverty eradication, rural development

## Abstract

**Introduction:**

This study explores the relationships between local governance effectiveness, population densities of Scheduled Castes (SC) and Scheduled Tribes (ST), and rural multidimensional poverty in India, using data from the 2011 Indian Census, 2011 Socio-Economic and Caste Census (SECC), 2020 Mission Antyodaya, and 2019/21 National Family and Health Survey (NFHS).

**Methods:**

The research examines how SC/ST population densities and local governance effectiveness are associated with the Multidimensional Poverty Index (MPI) across Indian states through regression and correlation analysis.

**Results:**

The study’s findings reveal a national, rural MPI of 0.110, with 26% of the rural population experiencing multidimensional poverty. The study’s results also show that a statistically significant negative correlation exists between rural MPI and local governance effectiveness, with a 0.32% reduction in MPI for every 1% improvement in governance effectiveness. Conversely, higher SC and ST population densities are associated with increased MPI, with a 0.14% rise in MPI for each 1% increase in these densities. The study also highlights that ST population density has a stronger association with MPI than SC population density, indicating greater vulnerability to multidimensional poverty in areas with higher ST populations.

**Discussion:**

Overall, the study underscores the importance of effective local governance in reducing rural poverty and suggests targeted efforts in areas with high SC and ST densities, particularly ST, to alleviate poverty. It also emphasizes the need for up-to-date data to understand and address rural poverty comprehensively.

## 1 Introduction

With about 65% of Indians residing in rural areas (Prasad et al., 2022; Ministry of Finance, 2023), poverty in India has historically been concentrated in rural areas (Datt et al., 2020), particularly among rural households belonging to scheduled castes and tribes (Gang et al., 2008). Therefore, the Indian government has made concerted efforts to eliminate poverty in rural areas, especially among backward castes through the delivery of several public services and implementation of a plethora of government schemes (Saxena et al., 2003; Yesudian, 2007; Radhakrishnan et al., 2018; Angom and Viswanathan, 2022). The vehicle through which these government efforts reach the target population in India is the local government or *gram panchayat* (Chathukulam et al., 2021). Gram panchayats are constitutionally mandated local self-government institutions at the village level in India, established under Article 243G of the Indian Constitution (Press Information Bureau (PIB), 2023). These democratic institutions serve as the primary administrative units in rural areas, typically covering a population of 5,000–15,000 people (Ministry of Panchayati Raj, 2020). Gram panchayats are responsible for implementing development programs, managing local resources, and delivering essential public services. They represent the lowest tier of India’s three-tier system of local governance (Panchayati Raj Institutions), functioning as crucial intermediaries between higher government levels and rural communities (Ministry of Panchayati Raj, 2020). Recent studies highlight their pivotal role in achieving sustainable development goals (SDGs) in rural India, particularly in areas such as poverty alleviation, education, and health (United Nations Development Program (UNDP), 2021).

**Figure 1:**
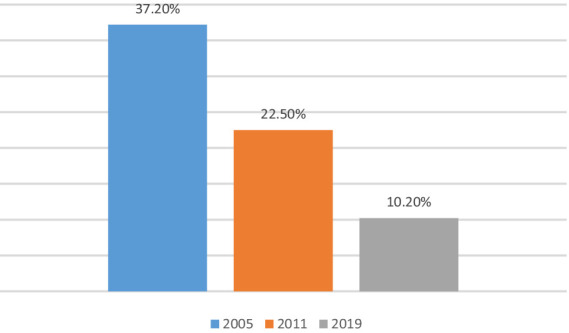
Trends in monetary poverty in India (2005–2019). Source: Visualization based on Planning Commission, Government of India (2009) and Sinha Roy and Van Der Weide (2022).

**Figure 2:**
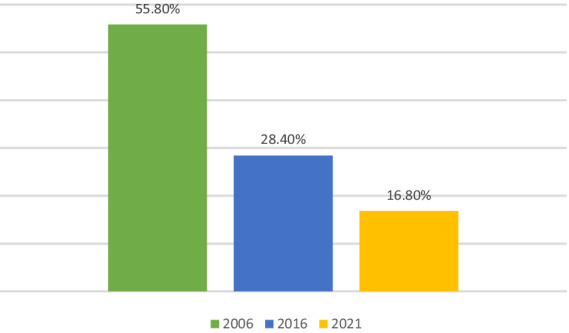
Trends in multidimensional poverty in India (2006–2021). Source: Visualization based on Das et al. (2023).

However, studies from the past two decades show that monetary and multidimensional poverties had declined drastically across Indian states, especially in rural areas and among lower caste populations (Planning Commission, Government of India, 2009; Alkire and Seth, 2015; Alkire et al., 2020; NITI Aayog, 2021; NITI Aayog, Government of India, 2021; Sinha Roy and Van Der Weide, 2022; Das et al., 2023; NITI Aayog, 2023). For example, Figure 1 shows that the incidence of monetary poverty fell from 37.2% in 2005 to 10.2% in 2019 (Planning Commission, Government of India, 2009; Sinha Roy and Van Der Weide, 2022). Also, Figure 2 shows that the incidence of multidimensional poverty fell from 55.8% in 2006 to 16.8% in 2021 (Das et al., 2023). Previous studies further state that the key factors influencing these drastic reductions in monetary and multidimensional poverties in India are economic growth and social protection programs, respectively (Borga and D’ambrosio, 2021; Singh, 2022).

Nonetheless, the gap in many of these studies is that they fail to highlight the factors contributing to these tremendous poverty reductions in rural India, especially if the government efforts at the local level, through the *gram panchayats*, are instrumental in the poverty reductions in rural areas and among backward castes. The ignoring of local government efforts in rural poverty eradication in previous literature is a global issue that exists due to the paucity of local government performance data (Lobao et al., 2012). To address this data issue and ensure accountability at the local government level, the Indian government initiated *Mission Antyodaya*, which is a “big data analysis” platform capturing the performance of *gram panchayats*, in 2017 (Chathukulam et al., 2021). Given the relative newness of the *Mission Antyodaya* platform, this gap in literature remains unaddressed in the Indian context. Also, the poverty measure that the current study focuses on is the multidimensional poverty index (MPI) proposed by Alkire and Santos (2010), which has gained traction globally, and in India due to its usefulness in tracking the first sustainable development goal—SDG 1 (NITI Aayog, 2023), entailing the total elimination of poverty by 2030 (Manikutty et al., 2023). This is because: (1) the MPI is a comprehensive measure of poverty, which considers multiple deprivations (e.g., health, education, and living standards) rather than just income and offers a more holistic understanding of poverty, especially in contexts like India where there are technical lapses in the data and methodology to be used for monetary poverty measurement (Edochie et al., 2022; Sinha Roy and Van Der Weide, 2022; Vollmer and Alkire, 2022), and (2) the *gram panchayats* focus on provisioning social services, and many of these services directly align with the indicators of multidimensional poverty (Chathukulam et al., 2021). Therefore, it will be useful to understand the extent to which the *gram panchayat’s* efficiency in provisioning these facilities impacts rural poverty reduction. Addressing this issue promises to improve the pro-poor policy making strategies for rural areas in India and in developing countries having similar characteristics as India; the lessons will also be valuable for dissimilar geographic contexts.

Notwithstanding, using other socio-economic factors, a few scholars have attempted identifying the factors driving rural and caste-wise poverty reduction. For example, Gupta (2022) explored the role of social protection in driving multidimensional poverty reduction in rural India and found that, although access to social protection programs reduces multidimensional poverty, it neither enables households to exit poverty nor cushions them against entry into poverty. Similarly, Biswal et al. (2023) investigated whether women’s empowerment drove multidimensional poverty reduction in rural Odisha and found that women’s age, occupation, and education status aided multidimensional poverty reduction. Another study (Gang et al., 2008) explored the factors driving the differences in poverty between scheduled caste (SC) and scheduled tribe (ST) households. The study found that the differences in the poverty incidences in SC and ST households stem from disparities in educational attainment and occupational structure (Gang et al., 2008). Also, Borooah et al. (2014) studied whether caste and religion were the basis for poverty and inequality in India, and found that people belonging to lower castes (SC and ST), as well as those who were Muslims faced higher poverty and inequalities than the rest of the population.

While all these factors highlighted in the literature are relevant for reducing multidimensional poverty, the current study focuses on the triune relationship between multidimensional poverty, local governance effectiveness, and SC/ST population density in the study area (i.e., rural India) for two reasons. First, there is scarcely any study on the role of governance, especially at the local level, in driving multidimensional poverty reduction in India, despite the overwhelming evidence in the literature that good multilevel governance is paramount for all aspects of economic development, including poverty reduction (Sadanandan, 2012; Jindra and Vaz, 2019). Second, most studies compare the proportion of SC/ST population among the poor population to that of other upper castes. But they fail to highlight whether having higher SC/ST populations is synonymous with higher poverty levels at the state level (e.g., Sundaram and Tendulkar, 2003; Panagariya and More, 2014). They also fail to show the poverty differences in between geographies having high SC populations and those having high ST populations (Borooah, 2005).

Against this backdrop, the objective of this paper is to examine how local governance effectiveness and SC/ST population density relate to multidimensional poverty in rural India. To the best of our knowledge, this is the first study: (1) to assess the relationship between local governance effectiveness, caste, and multidimensional poverty in rural India, and (2) to explain caste-based poverty disparities with population density data. We use the 2020 *gram panchayat* performance scores from the *Mission Antyodaya* platform to capture local governance (or local government effectiveness). To measure rural multidimensional poverty, we derive poverty data from the National Family and Health Survey (NFHS) for 2019/21. To estimate SC/ST population density at the rural level, we obtain SC/ST population data from the 2011 Socio-Economic and Caste Census (SECC) of India and data on land area from the 2011 Census of India. The reason for choosing the 2011 census and SECC data for measuring population density is that the two data sets are yet to be updated. Lastly, we use correlation and regression analysis to show the relationships between our choice variables. The rest of this paper is organized as follows. Section two presents the literature review. Section three gives the data used and methodology adopted for the analysis. Section four reveals and discusses the findings. Section five concludes the paper and offers relevant policy recommendations.

## 2 Literature review

While there are several definitions of poverty, the current study focuses on multidimensional poverty due to reasons based on the literature, which we expound on in section 2.1. Also, we attempt to understand the relationships between rural, multidimensional poverty and local governance effectiveness. To achieve that, we explore the literature on local governance effectiveness to identify the indicators or indices used for estimating its efficiency and to highlight its role in rural development, particularly poverty reduction, in section 2.2. Lastly, we explore the caste dimension to rural poverty in section 2.3.

### 2.1 Why multidimensional poverty?

For decades, poverty was seen as deprivations in many aspects of living, which can be lumped into and represented by one “key” deprivation indicator, usually monetary in character (Spicker, 2007). But with the seminal works of Narayan et al. (2000) and Sen (2005), alongside cross-country empirical evidences stemming from the Millennium Development Goals (MDGs) (Alkire and Santos, 2009; Alkire and Sumner, 2013), there is now a consensus in literature that poverty has multiple aspects that should be captured with multidimensional poverty measures (Thorbecke, 2013). Although many scholars have proposed various indices for estimating multidimensional poverty (e.g., Tsui, 2002; Bourguignon and Chakravarty, 2003; Jayaraj and Subramanian, 2010), the generally accepted index is the multidimensional poverty index (MPI), proposed by Alkire and Santos (2010), because it makes the best use of the data available in most countries (Vollmer and Alkire, 2022). The MPI is also preferred over other multidimensional poverty measures because it is estimated using the Alkire-Foster method, which allows the measure to reveal the multiple socio-economic issues that poor people face simultaneously, as opposed to principal component analysis adopted by previous scholars (Alkire and Santos, 2010). Owing to this, we adopt the MPI as the multidimensional poverty measure for this study.

### 2.2 Local governance effectiveness and poverty reduction

While researchers have not reached a consensus on what governance should entail, the World Bank has outlined three key components that succinctly describe the quality of governance across countries (Jindra and Vaz, 2019). They are: (1) the credibility of the government’s election process, (2) the efficiency and effectiveness of the government in formulating and implementing public policies, and (3) the trust of the citizenry in the government (Holmberg et al., 2009). Although several studies have attempted to comprehensively capture the impact of all aspects of governance on economic development and poverty reduction (Kwon and Kim, 2014; Hassan et al., 2020; Coccia, 2021; Nguyen et al., 2021), scholars disagree with an all-inclusive measure of governance because there are no clear boundaries in the definition of what governance entails (Fukuyama, 2016). Therefore, most economic and development studies focus on the second quality of governance component, which is often tagged as good governance (e.g., Sharma, 2007; Gisselquist, 2012). In other words, good governance is when the government can implement sound policies and deliver effective services to the public (Fukuyama, 2016; Rose-Ackerman, 2017).

Many studies argue that good governance is relevant for addressing multiple economic challenges, mainly poverty (Kwon and Kim, 2014; Hassan et al., 2020; Coccia, 2021; Nguyen et al., 2021). However, in the context of developing countries like India, some studies assert that the role of governance in economic development and poverty eradication is more effective when there is decentralization of power (Guevara, 2000; Shin, 2001; Chygryn et al., 2018). In economics and development literature, the term “decentralization of power’ is often used to highlight the devolution of power (Rodríguez-Pose and Gill, 2005), which is the transfer of public responsibilities to the local government (Rondinelli, 2006).

Various studies have explored the role of effective local governance in explaining multiple aspects of economic development and poverty alleviation in rural areas, but the findings on this relationship is mixed. For instance, some studies aver that local governments are instrumental in poverty reduction at the rural level (Mogale, 2005; Lobao et al., 2012), because, they possess first-hand information on local problems due to their closeness with localities, making them better qualified to formulate practical, context-based pro-poor policies (Sadanandan, 2012). But, other studies argue that higher poverty rates hinder local governments from achieving socio-economic development in rural communities (Lobao and Kraybill, 2009). There are also a few more studies that contend that the nexus between local governance and rural poverty reduction is complex (Steiner, 2007). Owing to these different findings, there has yet to be any consensus in literature on the instrumentality of local governments in spurring economic development and catalyzing poverty reduction. Therefore, the current study uses the Indian, rural context to weigh in on the debate.

### 2.3 Caste and poverty

In India, caste refers to the *varna* and *jati* classifications (Deshpande, 2001). By definition, *varna* refers to universal and hierarchal classification of caste according to occupational structure and *jati* refers to the different caste groups belonging to each category (Beteille, 1996; Deshpande, 2015). While *jati* comprises of over 2,000 subgroups, the *varna* comprises of five broad groups namely: “*Brahmins* (priest and teachers), *Kshatriya* (warriors and nobles), *Vaisya* (traders, merchants, and money lenders), *Sudras* (those doing menial jobs), and *Ati Sudra* (those engaged in the most odious menial jobs)” (Deshpande, 2001, 2015). Unlike the perception that *jati* is a subcategory of *varna, varna* is “a fluid scale over which *jatis* try to align themselves” (Deshpande, 2015). Since the “inclusion of caste” into the 2011 Indian Census, there has been debates on whether caste plays a significant role in social and economic stratification in modern India (Desai and Dubey, 2012). Despite the growing arguments, various social and economic studies find that caste is still associated with the socioeconomic and poverty statuses of individuals and households in India.

For example, Borooah (2005) used data obtained by the National Council of Applied Economic Research (NCAER) in 1994 to investigate inequality and poverty in India through the lenses of caste discrimination. The study found that, in comparison to other upper castes, people belonging to SC/ST castes were more vulnerable to poor health, low educational attainments, landlessness or marginal land ownership, social discrimination, and poor economic conditions. Similarly, Kumar (2014) used the National Sample Survey Organisation’s (NSSO) data to study the changes in the access to basic amenities in rural and urban India from 1993 to 2008. The study revealed that, when compared to other caste groups, there was low improvement in the access to basic services among the SC/ST populations in both rural and urban areas. Another study by Lastrapes and Rajaram (2016) used the National Family and Health Survey (NFHS) data for 2005–06 to examine the role of gender and caste in Indian poverty. They found that female headed households and households belonging to lower castes were the poorest groups and belonging to a lower caste household had a stronger impact on poverty than belonging to a female headed household. Many other studies (Nayar, 2007; Thorat, 2010; Mosse, 2018; Narendranath et al., 2018; Pradhan et al., 2022) have reported similar findings.

But, the gap in these studies is that they have not addressed the relationship between caste and poverty using state-wide caste population density data. Owing to that, this study attempts to find out if states with higher backward caste populations have higher poverty ratios and if there is any difference in the state-wise poverty levels when the SC population is higher than ST population and vice versa.

## 3 Data and methods

### 3.1 Dependent variable

The dependent variable for this study is multidimensional poverty. As expressed in section two, the poverty measure adopted for this study is the multidimensional poverty index (MPI), which was propounded by Alkire and Santos (2010). The MPI is a poverty measure that reflects the various deprivations that poor people face at the same time (Alkire and Santos, 2010). It comprises of three dimensions and 10 indicators (Alkire et al., 2020), as shown in Table 1. Mathematically, the MPI is a product of the incidence of poverty, denoted by H, and the intensity of poverty, denoted by A (Alkire and Seth, 2015). The incidence of poverty captures the proportion of people that are poor in a given population or sample while the intensity of poverty captures the average deprivation score of poor people (Alkire and Santos, 2014). For further details on the derivation of H, A, and MPI, see Alkire et al., 2015. In literature, several authors have estimated the MPI using the Indian Human Development Survey (IHDS), the National Sample Survey Organisation (NSSO), and the National Family and Health Survey (NFHS) data sets (Alkire and Seth, 2015; Alkire et al., 2020; Das et al., 2021; Dehury and Mohanty, 2015; Mothkoor and Badgaiyan, 2021). But, NFHS data is the most favored in literature (Das et al., 2021). Hence, we calculate the MPI for rural areas across Indian states and union territories using the 2019/21 NFHS data (i.e., the most recent data).

**Table 1:** Dimensions and indicators of the multidimensional poverty index.

Dimensions	Weights (per dimension)	Indicators	Weights (per indicator)	Deprivation cut-off (Deprived if…)
Health	1/3	Nutrition	1/6	Anyone below 70 years of age, for whom there is nutritional information, is malnourished.
		Child Mortality	1/6	A child under 18 years of age has died in the family in the five-year period preceding the survey.
Education	1/3	Years of Schooling	1/6	Not even one member of the household aged 10 years or older has completed six years of schooling.
		School Attendance	1/6	Any school-aged child is not attending school up to the age at which he/she would complete class 8.
Living Standards	1/3	Cooking Fuel	1/18	A household cooks with dung, agricultural crops, shrubs, wood, charcoal, or coal.
		Sanitation	1/18	The household has unimproved or no sanitation facility or it is improved but shared with other households.
		Drinking Water	1/18	The household does not have access to improved drinking water or safe drinking water is at least a 30-min walk from home (as a round trip).
		Electricity	1/18	The household has no electricity.
		Housing	1/18	The household has inadequate housing: the floor is made of natural materials, or the roof or wall are made of rudimentary materials.
		Assets	1/18	The household does not own more than one of these small assets: radio, TV, telephone, computer, animal cart, bicycle, motorbike, or refrigerator; and does not own any big asset such as car or truck.

Source: Alkire et al. (2018).

### 3.2 Independent variables

The independent variables we use in this study are local governance effectiveness and caste. We represent the first independent variable—local governance effectiveness—with the average *gram panchayat* performance scores for each state and union territory. The average *gram panchayat* performance score is derived from the Gram Panchayat Development Index (GPDI), developed by the Ministry of Panchayati Raj, Government of India, as a measure of local governance effectiveness (Ministry of Panchayati Raj, 2018). Introduced in 2018, the GPDI evaluates gram panchayat performance across six thematic areas: health, nutrition, and sanitation; economic development and livelihood; financial inclusion; access to basic facilities; infrastructure development; and women’s empowerment (Chathukulam et al., 2021). The index is constructed using administrative data collected annually through a standardized assessment framework (Ministry of Panchayati Raj, 2018). While the GPDI provides a systematic measure of local governance performance, it is important to acknowledge its limitations. For instance, the index may not fully capture the nuances of governance quality, and the data collection process could be subject to reporting biases (World Bank, 2022). Additionally, the standardization of metrics across diverse regional contexts presents methodological challenges that should be considered when interpreting results (United Nations Development Program (UNDP), 2021).

Despite these gaps, the current study employs the GDPI as the proxy for local governance effectiveness because it still captures the efficiency and adequacy of the *gram panchayat* in addressing rural development issues to a large extent (Chathukulam et al., 2021). The maximum GDPI score per panchayat is 100 (Chathukulam et al., 2021). This implies that, the closer the average *gram panchayat’s* score to 100, the more efficient the *gram panchayat*. We obtain the *gram panchayat* scores for each state in 2020 from the Mission Antyodaya database. Further, we estimate the second independent variable—caste—using the SC/ST population density per state. To calculate the population density, which is the quotient of total population and land area, we obtain state-wise rural land area from the latest Indian census (i.e., the 2011 census) data and total SC/ST population per state from the latest Indian socioeconomic and caste census (SECC) data—that is, the 2011 SECC data.

### 3.3 Strategies for data analysis

The current study attempts to observe the role of local governance effectiveness and SC/ST population density in the prevalence of multidimensional poverty in rural areas across Indian states. Given that our data is cross-sectional, we use correlation and regression models for data analysis (Setia, 2016). To measure the “strength and direction” of three pairs containing two variables each (i.e., the association of SC population density and multidimensional poverty, ST population density and multidimensional poverty, and local governance effectiveness and multidimensional poverty), we use Pearson’s correlation coefficient (Ahlgren et al., 2003). We also use a regression framework to examine the extent to which multidimensional poverty is explained by our choice variables—local governance effectiveness and SC/ST population density (Balasubramanian et al., 2023). With LG representing local governance effectiveness, $SC_{P}$ representing scheduled caste population density, $ST_{P}$ representing scheduled tribe population density, and ε denoting the error/residual term, the regression equation for this study is:


$$MPI=\beta_{0}+\beta_{1}\left(LG\right)+\beta_{2}\left(SC_{P}\right)+\beta_{0}\left(ST_{P}\right)+\varepsilon$$

Furthermore, we analyse and visualize our data using excel, SPSS, and python.

### 3.4 Inclusion and exclusion criteria

To handle the problem of missing values in this study, we adopt the listwise deletion method, which is the most common approach for dealing with missing values in literature (Kang, 2013). The listwise deletion method entails that we exclude observations (i.e., states, in this case) lacking complete data for either the dependent variable or any of the independent variables from our study (Kang, 2013). Therefore, out of the 28 states and 8 union territories in India (hereafter referred to as 36 geographic entities), we include only 29 geographic entities and exclude 7, namely: Chandigarh, Delhi, Ladakh, Lakshadweep, Puducherry, Arunachal Pradesh, and Telangana.

## 4 Results and discussion

### 4.1 Descriptive statistics

Table 2 presents the descriptive statistics of our data used for this study. The descriptive results show that the average rural MPI across states is 0.088, with the minimum and maximum values being 0.008 and 0.203, respectively. Similarly, the average value for local government performance is 39.4, with the minimum and maximum values being 22 and 67.5, respectively. In terms of population density, the average SC and ST population densities in rural India are 10 and 9 people per square kilometers, respectively. From the standard deviation values, we observe that there are higher disparities across states in SC population density, followed by ST population density, local governance effectiveness, and rural MPI.

**Table 2:** Descriptive statistics (*N* = 29).

Statistic	Rural MPI	Local governance	SC population density	ST population density
Mean	0.0877	39.4458	10.0208	9.2965
Standard deviation	0.0537	9.6409	12.3007	11.9227
Minimum value	0.0084	21.9595	0	0.0033
Maximum value	0.2028	67.4527	53.6125	60.4002

Source: Authors’ calculation.

### 4.2 MPI, local governance effectiveness, and SC/ST population in rural India

Table 3 shows that the values for *MPI*, *H*, and *A* in rural India are 0.110, 0.260, and 0.424, respectively. This implies that the 26% of multidimensionally poor Indians living in rural areas are deprived in 42.4% of the multidimensional poverty indicators, which is equivalent to a full deprivation with respect to the dimensions of either health or education, and in one indicator of living standard. The states with the lowest and highest prevalence of rural multidimensional poverty are Kerala and Bihar, respectively. This finding matches with that of Alkire et al. (2020), who found that multidimensional poverty was highest in Bihar and lowest in Kerala, and slightly departs from that of Das et al. (2023), who found that multidimensional poverty was highest in Jharkhand and lowest in Kerala. For local governance effectiveness, Table 3 reveals that the average performance of *gram panchayats* in India was 39.4 out of 100 in 2020. This implies that local governance effectiveness is barely effective in rural India. The states with the best *gram panchayat* performance in India were Kerala and Gujarat, while Manipur and Meghalaya had the worst performing *gram panchayats*. Furthermore, while Nagaland, Andaman and Nicobar Island, Mizoram, and Meghalaya had the lowest SC population density, West Bengal had the highest SC population density. Similarly, while Punjab and Haryana had the lowest ST population density, Dadra & Nagar Haveli and Daman & Diu had the highest ST population density.

**Table 3:** Rural multidimensional poverty, local governance effectiveness, and rural SC/ST population density.

States	Rural multidimensional poverty (2019/21)			Gram panchayat performance scores (GPPS)	Rural SC/ST population density for 2011 (People per Square Kilometer)	
	MPI	H	A	GPPS 2020	SC	ST
Jammu & Kashmir	0.0403	0.101	0.400	36.1	1	1
Himachal Pradesh	0.036	0.093	0.389	35.9	5	1
Punjab	0.035	0.087	0.403	39.4	25	0
Uttarakhand	0.058	0.148	0.392	33.8	5	1
Haryana	0.056	0.134	0.416	45	16	0
Rajasthan	0.117	0.280	0.419	35.3	6	5
Uttar Pradesh	0.139	0.323	0.429	35.7	27	1
Bihar	0.203	0.453	0.448	37.2	33	3
Sikkim	0.028	0.073	0.381	40.5	1	5
Nagaland	0.116	0.293	0.396	27.3	0	16
Manipur	0.094	0.233	0.405	22	1	10
Mizoram	0.058	0.136	0.428	39.5	0	5
Tripura	0.110	0.264	0.417	45.1	13	26
Meghalaya	0.174	0.379	0.458	24.6	0	20
Assam	0.116	0.276	0.422	30.5	6	11
West Bengal	0.112	0.274	0.409	40.5	54	14
Jharkhand	0.193	0.443	0.436	30	8	19
Odisha	0.130	0.303	0.427	32.8	10	14
Chhattisgarh	0.130	0.304	0.428	37.6	5	13
Madya Pradesh	0.152	0.360	0.422	33.8	6	10
Gujarat	0.108	0.260	0.413	59.7	2	8
Dadra & Nagar Haveli and Daman & Diu	0.068	0.177	0.385	49.9	3	60
Maharashtra	0.071	0.178	0.400	37.9	6	6
Andhra Pradesh	0.054	0.135	0.397	45.3	6	2
Karnataka	0.059	0.149	0.398	39.5	8	4
Goa	0.012	0.032	0.381	47.4	1	10
Kerala	0.008	0.022	0.376	67.5	21	3
Tamil Nadu	0.032	0.086	0.377	48.3	22	2
Andaman & Nicobar Islands	0.033	0.086	0.385	45.8	0	1
All India (Rural)	0.110	0.260	0.424	39.4	11	6

Source: Authors’ calculation.

### 4.3 The interplay of multidimensional poverty, local governance effectiveness, and SC/ST population density in rural India

#### 4.3.1 Description of correlation results

The results obtained from Pearson’s correlation matrix reveal that the MPI has a statistically significant negative correlation with local governance effectiveness, and positive correlation with SC and ST population densities. However, we proceed with interpreting the results because evidence from the literature shows that a weak statistical significance (as is the case of MPI versus SC/ST population densities) neither negates the reality or importance of the relationship between variables nor implies that the relationship between the variables is absent (Kafi and Ansari-Lari, 2022). Studies suggest that, in lieu of the *p*-value, other statistical measures of evidence (such as confidence interval, r-squared, odds ratios, correlation coefficients, and regression coefficients) can be used to analyse the relationship between variables (Aarts et al., 2012; Kafi and Ansari-Lari, 2022). Of the statistical measures of evidence listed, the confidence interval is said to provide more accurate insights than the *p*-value (Aarts et al., 2012). Therefore, we discuss our findings using the correlation coefficients obtained and the confidence intervals derived.

From Table 4A, it is evident that MPI’s correlation with ST population density was higher than with SC population density, even though both correlations are weak. This means that although states having higher SC/ST populations will most likely have higher rural multidimensional poverty, states with more ST than SC populations seem to be more vulnerable to rural multidimensional poverty. Also, the confidence intervals of the relationship between MPI and SC/ST population densities fall within 95%. Hence, we are 95% confident that the proportion of SC/ST dominated states having high, rural multidimensional poverty will always fall within the intervals given in Table 4B. However, we further validate this finding with our regression results presented in section 4.3.2, as suggested in literature (Aarts et al., 2012).

**Table 4:** Correlation among variables.

(A) Pearson’s correlation coefficients				
	MPI	Local governance effectiveness	ST density	SC density
MPI	1			
Local Governance Effectiveness	−0.518***	1		
ST Den	0.261	−0.006	1	
SC Den	0.168	0.183	−0.144	1
***p < 0.01. Source: Authors’ calculation.				

(B) Confidence Intervals (CI)	
Relationships	CI
MPI versus LG	−0.5224, −0.5131
MPI versus SC Density	0.1617, 0.1741
MPI versus ST Density	0.2555, 0.2674
LG versus SC Density	0.1773, 0.1896
LG versus ST Density	−0.0119, 0.0008
SC Density versus ST Density	−0.1503, −0.1378

Source: Authors’ calculation.

Table 4A also shows that rural multidimensional poverty has a moderate negative correlation with local governance effectiveness, and this correlation is significant at 1% level. This implies that states having more effective and well performing *gram panchayats* will likely have lesser rural multidimensional poverty than their counterparts. This finding is in line with that of Mogale (2005) and Lobao et al. (2012). When we observe the relationship between local governance effectiveness and SC/ST population density, it is found that there is no correlation between ST population density and local governance effectiveness. Meanwhile, there is a slight positive correlation between SC population density and local governance effectiveness, implying that states with higher SC population density are more likely to have better performing *gram panchayats*. This could be why poverty is lesser among SCs than STs, as supported by literature (Gang et al., 2008).

**Figure 3:**
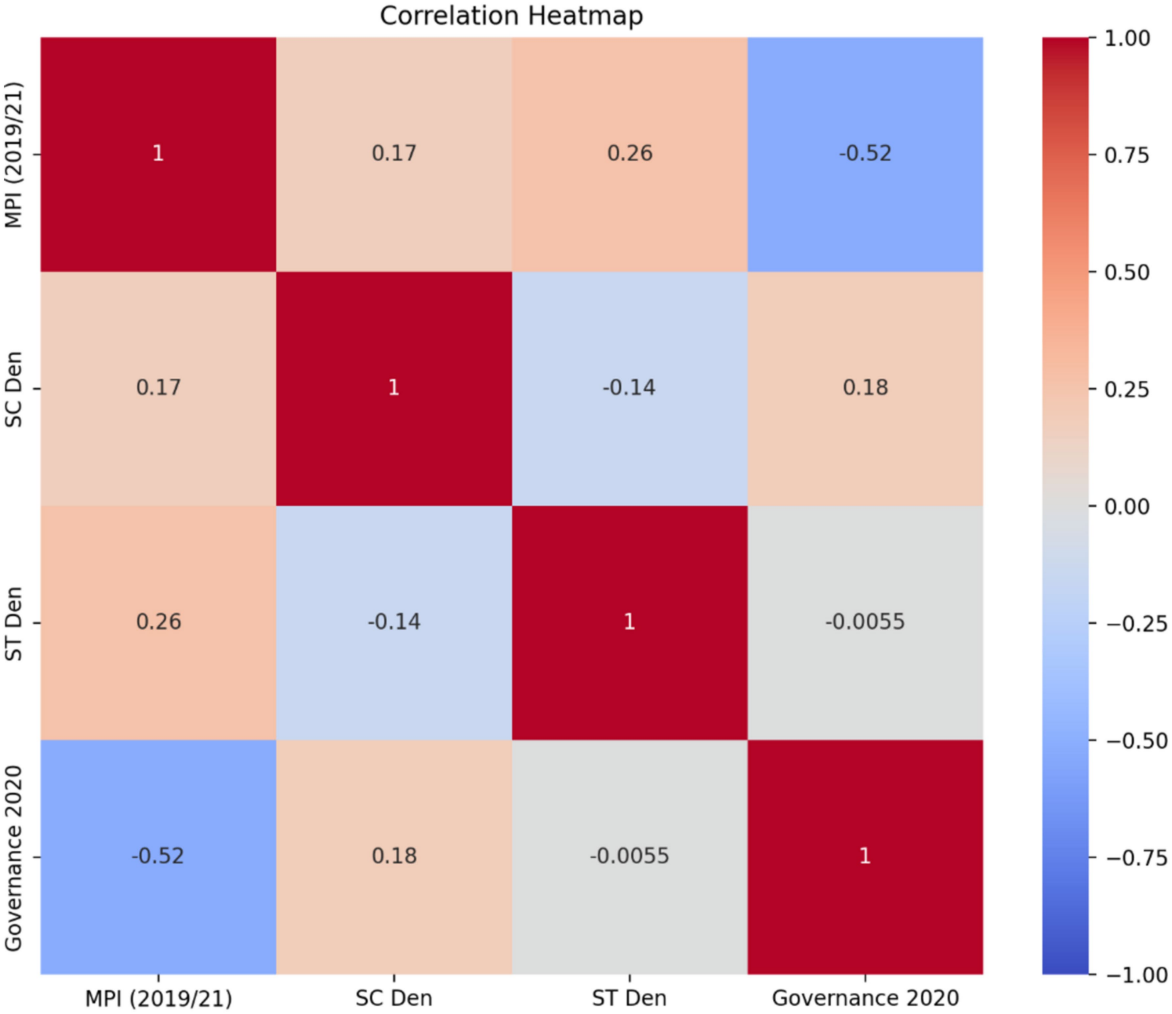
Pearson’s correlation heat map. Source: Authors’ calculations.

In the heat map, shown in Figure 3, positive correlation was represented using the auburn color (or brownish orange color) while negative correlation was represented with blue color. Higher positive or negative correlations were depicted through darker shades of the respective colors while lower positive or negative correlations were depicted through lighter shades of the respective colors. Given that lighter shades of both auburn and blue dominate the heat map, it implies that the correlation among our variables majorly tended to be medium or low. By visually representing the correlation matrix, the heat map confirms the observations made earlier about the relationships between our choice variables and clarifies the strength of those relationships.

#### 4.3.2 Discussion of regression results

From the regression analysis conducted (see Table 5A), we observe that the probability of an *F*-statistic (Prob > *F*) is 0.0025, which implies that the overall model is statistically significant and our independent variables are reliable enough to predict the dependent variable. This result is further supported by the model’s R-squared value, which is 0.430, thus indicating that about 43% of the variance in rural multidimensional poverty is explained by local governance effectiveness and SC/ST population density. Table 4 shows that the baseline MPI or the constant is approximately 0.187, which is statistically significant at 1% level. This means that in the absence of the choice variables, the mean value of the MPI will be 0.187.

**Table 5:** Relationship between MPI and choice variables.

(A) Model summary			
R Squared	Adjusted R Squared	*F*-Statistic	Prob (*F*-Statistic)
0.430	0.362	6.287	0.0025
Predictors: (Constant), Local Governance Effectiveness, SC Population Density, and ST Population Density.			
Source: Authors’ calculation.			

(B) Regression coefficients		
Variables	Coefficient	Standard error
Constant	0.187***	0.035
Local governance effectiveness	−0.0032***	0.001
SC population density	0.0014**	0.001
ST population density	0.0014*	0.001

***p < 0.01, **p < 0.05, and *p < 0.1.

Source: Authors’ calculation.

From Table 5B, we find that a 1% increase in SC population density will spur a 0.14 percent increase in rural multidimensional poverty, and this finding is statistically significant at 5% level. We also find that a 1% increase in ST population density will spur a 0.14 percent increase in rural multidimensional poverty, and this finding is statistically significant at 10% level. These two findings on the SC/ST population-MPI nexus reveals that rural multidimensional poverty is positively and significantly associated with the proportion of SCs and STs present in any geographic entity. This finding implies that geographic entities with higher SC/ST populations in rural areas should target their pro-poor policies and programs toward SCs and STs. While this is the first study to look at the relationship between multidimensional poverty and SC/ST population densities in rural India, our findings are similar to those of other studies that find that multidimensional poverty is higher among STs than SCs (Alkire and Seth, 2015; Alkire et al., 2020; Das et al., 2021; Das et al., 2023).

When we consider the relationship between rural multidimensional poverty and local governance effectiveness, we notice that a 1% increase in local government performance decreases rural multidimensional poverty by 0.32 percent, and this decrease is statistically significant at 1% level. The implication of our obtained rural MPI-local governance effectiveness nexus is that better governance is associated with lower MPI scores (which indicates less poverty). This finding substantiates our previous correlation results and aligns with the existing literature from other countries (Mogale, 2005; Lobao et al., 2012). So, our study contributes to literature by revealing that the poverty-local governance effectiveness nexus in rural India is inverse, which motivates the call for a more responsible leadership at the local level to catalyze poverty reduction, especially in regions with high populations of backward castes.

**Figure 4:**
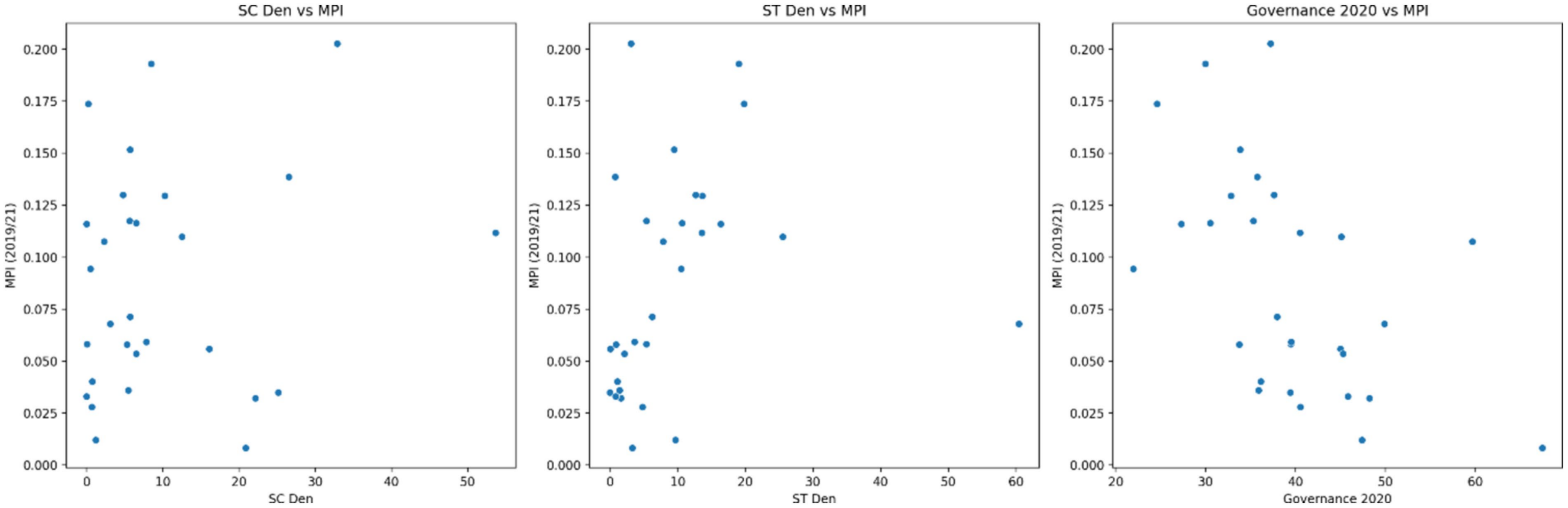
Scatter plots showing the relationships between the choice variables. Source: Authors’ calculation.

**Figure 5:**
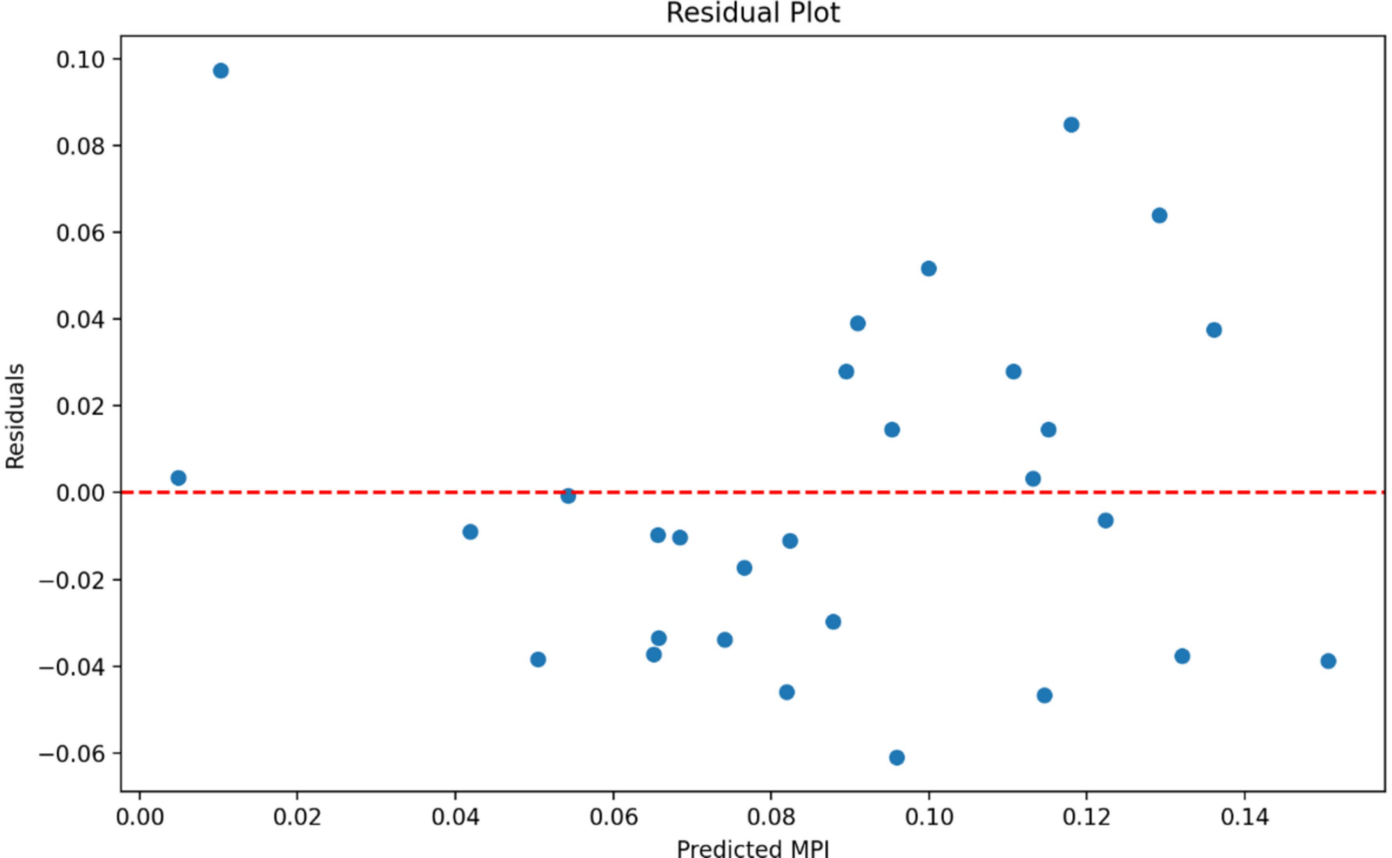
The residual plot. Source: Authors’ calculation.

The scatter plots in Figure 4 show the relationships between each independent variable and the MPI. Of the three independent variables employed in this study, the Governance 2020 plot (or local governance effectiveness) shows the strongest association with the MPI. Also, the residual plot in Figure 5 shows a relatively random scatter of points around the horizontal line at *y* = 0, which suggests that the linear regression model’s assumptions are reasonably met. However, there’s a slight pattern visible, indicating that the model might not capture all the relationships in the data. Therefore, we suggest that future studies adopt more robust and complex analyses to rectify this limitation of our study.

#### 4.3.3 Cluster analysis

A cluster analysis was done and it identified three distinct groups of states based on MPI and related predictors, providing valuable insights into regional patterns of poverty (Table 6).

**Table 6:** Cluster means.

Cluster	MPI	SC Den	ST Den	Gram influence	Governance
0	0.1317	11.9714	11.8439	4.6275	33.7051
1	0.068	3.1155	60.4002	3.9857	49.8947
2	0.0451	8.5634	3.0988	4.0446	44.4401

***p < 0.01, **p < 0.05, and *p < 0.1. Source: Authors’ calculation.

**Cluster 0 (High MPI States):** This cluster includes 14 states, primarily from northern and eastern India, such as Bihar, Uttar Pradesh, and Odisha. These states have the highest MPI (0.1317), moderate SC Den (11.97), moderate ST Den (11.84), and the lowest Governance score (33.71). The high MPI in these states reflects persistent socio-economic challenges, including weaker governance and higher poverty levels. These states require focused policy interventions to address structural inequalities and improve governance mechanisms (Table 7).

**Table 7:** Number of states in each cluster.

Cluster	Count
0	14
1	1
2	14

***p < 0.01, **p < 0.05, and *p < 0.1. Source: Authors’ calculation.

**Cluster 1 (Unique Case):** This cluster consists of a single region, Dadra & Nagar Haveli and Daman & Diu. It is characterized by moderate MPI (0.068), the lowest SC Den (3.12), the highest ST Den (60.40), and the highest Governance score (49.89). The unique demographic composition of this region, with a high concentration of Scheduled Tribes, sets it apart from other clusters. Tailored policies addressing tribal welfare and leveraging strong governance structures could further reduce poverty in this region.

**Cluster 2 (Low MPI States)**: This cluster includes 14 states, primarily from southern and western India, such as Kerala, Tamil Nadu, and Gujarat. These states have the lowest MPI (0.0451), moderate SC Den (8.56), the lowest ST Den (3.10), and good Governance scores (44.44). The low MPI in these states reflects better socio-economic conditions, stronger governance, and effective local institutions. These states serve as models for poverty reduction strategies that can be adapted to other regions.

The findings from both analyses highlight the complex interplay between demographic factors, governance, and poverty. The non-linear relationship between SC Den and MPI suggests that interventions should be tailored to the specific demographic composition of regions, with a focus on areas where the marginal impact of SC Den is most significant. The clustering analysis underscores the importance of regional patterns, with high MPI states requiring targeted governance reforms and socio-economic investments, while low MPI states can serve as benchmarks for best practices.

#### 4.3.4 Further analysis on the poverty-governance nexus

Since the previous results show that local governance effectiveness is more associated with multidimensional poverty reduction than caste, we attempt to determine the capacity of the gram panchayats in addressing the 10 aspects of multidimensional poverty using simple regression analysis. We present the regression results showing the relationship between each MPI indicator and local governance effectiveness in Table 8, from which it is evident that all r-squares are low. Nevertheless, we proceed with interpreting the relevant models by following the recommendations of Ozili (2023), which entails that models can be interpreted as long as the low r-squared value falls within 0.10–0.50 and the independent variable or at least one of the independent variables included in the model is statistically significant. Given this prerequisite, we find that local governance effectiveness plays a statistically significant role in addressing poverty deprivations related to housing, clean cooking fuels, and drinking water. This finding implies that, although *gram panchayats* are important for reducing multidimensional poverty as a whole, their expertise is likely directly associated with improvements in the rural populations’ access to quality housing, clean cooking fuels/facilities, and clean drinking water. Therefore, it is expedient that *gram panchayats* are fully equipped, trained, and monitored by higher levels of government to enable them to efficiently provide these specific services to rural areas.

**Table 8:** MPI indicators versus local governance effectiveness.

MPI indicator	R-squared	Constant	Regression coefficient	Standard error (constant)	Standard error (coefficient)
Drinking water	0.117	0.395***	−0.005*	0.099	0.002
Electricity	0	0.087	6.380E-5	0.058	0.001
Sanitation	0.001	0.515***	0	0.143	0.004
Housing	0.286	1.113***	−0.009***	0.112	0.003
Assets	0.026	0.520***	−0.003	0.136	0.003
Cooking fuel	0.197	1.057***	−0.006**	0.089	0.002
Years of schooling	0.005	0.419***	−0.001	0.071	0.002
School attendance	0.088	0.127***	−0.001	0.029	0.001
Nutrition	0.001	0.735***	0	0.059	0.001
Child mortality	0.063	0.204***	0.002	0.069	0.002

***p < 0.01, **p < 0.05, and *p < 0.1.

Source: Authors’ calculation.

## 5 Conclusions and policy recommendations

The present study attempted to explore the relationship between rural multidimensional poverty, local governance effectiveness, and SC/ST population density across Indian states. From the analysis, we obtain two key findings. The first is that local governance effectiveness has a highly, statistically significant negative relationship with rural multidimensional poverty in India. Also, among the independent variables used for the study, local governance effectiveness appears to have the strongest relationship with rural MPI, thus suggesting that states with better performing *gram panchayats* will mostly have lower rural multidimensional poverty. This finding has important policy implications. Firstly, it necessitates a careful examination of how *gram panchayats* function and their role in poverty alleviation, as their strong positive association with MPI might indicate that they may be facing implementation challenges, hence implying that there is a need for capacity building at the local governance level. Secondly, it suggests that while overall governance quality shows potential for poverty reduction, there might be a disconnect between local and broader governance structures that needs to be addressed. The results call for a comprehensive review of local governance mechanisms, particularly focusing on how *gram panchayats* can be better equipped and oriented toward effective poverty reduction strategies.

Given that poverty reduction in rural India is likely to be driven by effective *gram panchayats*, it is important that *gram panchayats* are closely monitored and supervised to ensure transparency and accountability in their efforts toward rural development and poverty eradication. Also, this implies that *gram panchayats* should be given full autonomy to both identify and resolve the challenges faced by the communities under their jurisdiction, which is currently not happening as the devolution of power to *gram panchayats* is unequal across states, thus inhibiting their effectiveness in carrying out their constitutional duties, especially in underdeveloped states (Kumar, 2019). Another issue that impedes the efforts of *gram panchayats* is political clientelism, where the local political elites try to pander to the regional political elites rather than those at the grassroots as a coping mechanism to maintain their political relations and positions, hence making the presence of *gram panchayats* to be counterproductive in driving socioeconomic changes and developments within communities (Kumar, 2019; Kashyap, 2022). This issue can only be resolved when *gram panchayats* are autonomous and empowered, which is yet to happen. So, we recommend that the government should ensure that *gram panchayats* have the complete power to bring their full potential to the table, in terms of rural policy making, so as to catalyze rural development and reduce multidimensional poverty.

The second is that geographic areas that are densely populated by SCs and STs are more likely to be prone to rural multidimensional poverty. The study’s results further show that high multidimensional poverty in rural areas is more associated with geographic areas that have more STs than SCs. This finding highlights the importance of understanding the nuances in the relationship between poverty and different backward castes. Although poverty is mostly present among all backward castes (i.e., SC and ST), the current study shows that it is more severe among the STs. This finding is in line with that of Vempati et al. (2020) on the economic and social challenges faced by the impoverished Musahar community of Ratanpur, which is an ST community located in the Indian state of Bihar. The implication of this finding is that there is a need for a more focused poverty targeting for areas densely populated by STs. One of the major reasons why SCs and STs, especially the latter, are more exposed to rural multidimensional poverty is because they face discriminations from their immediate and extended environments, thus excluding them from benefitting from government schemes and public services or even being aware of such socioeconomic opportunities (Development Monitoring and Evaluation Office (DMEO), NITI Aayog, Government of India, 2022). To eradicate multidimensional poverty in rural areas, the government needs to intensify their efforts in protecting regions comprising of many backward caste groups from social exclusion and information asymmetry, particularly in terms of government welfare programs. Also, for government welfare programs to be effective in reducing multidimensional poverty for these historically excluded groups, then they must be tailored to synchronize with the realities of these backward populations. Therefore, we emphasize the need for targeted interventions in areas with high SC populations.

Despite the two strong contributions of this study, there are three key limitations of our study. The first is that the population data used was for 2011 census. Given that more than 10 years have passed, the scenario may have changed as the period between 2011 and 2021 had also witnessed several developmental interventions aimed at poverty reduction. In this regard, we suggest that the new Census of 2021 (which is still ongoing) may incorporate the critical governance performance variables for better understanding of the MPI scenario leading to more targeted policy interventions. Although our regression model explains about 43% of the variance in MPI, which suggests that our choice variables are important, the second limitation of our study is that there are likely other variables not included in this analysis that also influence multidimensional poverty. Therefore, we suggest that future studies should identify those factors (e.g., economic growth, employment rate, women’s empowerment, et cetera) and address them. The last limitation of our study is that the relationships between the variables are not strongly linear, as evidenced by the residual plot and scatter plots. This suggests that more complex models or additional variables might be needed to better explain the variations in MPI.

## Data Availability

Publicly available datasets were analyzed in this study. This data can be found here: https://secc.gov.in/; http://www.censusindia.gov.in/; https://missionantyodaya.nic.in/; and https://dhsprogram.com/Data/.
